# 107 The Ecosystem Model of Five Solutions to Enhance Work and Functional Capacity Through Physical Activity in the Workplace

**DOI:** 10.1093/eurpub/ckae114.024

**Published:** 2024-09-26

**Authors:** Reetta Laakkonen, Miia Malvela

**Affiliations:** Adults on the move -program, Jyväskylä, Finland; Adults on the move -program, Jyväskylä, Finland

## Abstract

**Purpose:**

Broader solutions than the traditional approaches are needed to promote physical activity (PA) in the workplaces. To tackle this problem, the Finnish government funded program Adults on the Move (AMP) has collaboratively developed an ecosystem model consisting of five solutions to enhance work and functional capacity through PA in the workplace. The approach is new as it utilized the impact investing model developed by future fund Sitra. The ecosystem model has been developed for the future planning purposes to identify the points of influence PA has within the existing structures of Finnish work life.

**Project description:**

The model was developed collaboratively by a working group of various stakeholders including occupational health care, the Finnish Institute of Occupational Health, employers’ pension insurance, trade unions, employer advocacy organizations, and the Ministry of Social Affairs and Health.

The working group identified five impact pathways for PA within existing work life structures:

1. Society providing more incentives to employers, such as tax cuts, to promote PA and well-being.

2. A new way of assessing risks at workplaces, including both physically demanding and inactive work, with PA reducing risks in both.

3. More consistent promotion of PA in collaboration with occupational health care to enhance overall work capacity.

4. Improving psychological, physical, and social balance in the workplace through PA.

5. Providing more training to supervisors on work capacity management, including aspects of PA and recovery.

The work was evaluated by several stakeholders, including Finland’s largest business confederation, the Confederation of Finnish Industries, the Centre for Occupational Safety, The Central Organisation of Finnish Trade Unions, and the Ministry of Economic Affairs and Employment of Finland.

AMP prepared and disseminated the completed work to stakeholders. As a result, for example the Ministry of Social Affairs and Health is strengthening the role of PA in occupational health care, enhancing the competence of services related to workplace collaboration and individual work capacity improvement.

In conclusion, the impact investment model provides a useful framework to address the systemic problem of physical inactivity among working-age people. This novel approach to modeling the topic has been well-received among stakeholders.

